# A disulfidptosis-related lncRNAs signature in hepatocellular carcinoma: prognostic prediction, tumor immune microenvironment and drug susceptibility

**DOI:** 10.1038/s41598-024-51459-z

**Published:** 2024-01-07

**Authors:** Yanqiong Liu, Jiyu Meng, Xuelian Ruan, Fangyi Wei, Fuyong Zhang, Xue Qin

**Affiliations:** Key Laboratory of Clinical Laboratory Medicine of Guangxi Department of Education, Department of Clinical Laboratory, The First Affiliated Hospital of Guangxi Medical University, Nanning, Guangxi China

**Keywords:** Cancer, Gastrointestinal diseases, Functional genomics, Cancer, Computational biology and bioinformatics, Genetics, Immunology, Molecular biology, Biomarkers, Gastroenterology

## Abstract

Disulfidptosis, a novel type of programmed cell death, has attracted researchers’ attention worldwide. However, the role of disulfidptosis-related lncRNAs (DRLs) in liver hepatocellular carcinoma (LIHC) not yet been studied. We aimed to establish and validate a prognostic signature of DRLs and analyze tumor microenvironment (TME) and drug susceptibility in LIHC patients. RNA sequencing data, mutation data, and clinical data were obtained from the Cancer Genome Atlas Database (TCGA). Lasso algorithm and cox regression analysis were performed to identify a prognostic DRLs signature. Kaplan–Meier curves, principal component analysis (PCA), nomogram and calibration curve, function enrichment, TME, immune dysfunction and exclusion (TIDE), tumor mutation burden (TMB), and drug sensitivity analyses were analyzed. External datasets were used to validate the predictive value of DRLs. qRT-PCR was also used to validate the differential expression of the target lncRNAs in tissue samples and cell lines. We established a prognostic signature for the DRLs (MKLN1-AS and TMCC1-AS1) in LIHC. The signature could divide the LIHC patients into low- and high-risk groups, with the high-risk subgroup associated with a worse prognosis. We observed discrepancies in tumor-infiltrating immune cells, immune function, function enrichment, and TIDE between two risk groups. LIHC patients in the high-risk group were more sensitive to several chemotherapeutic drugs. External datasets, clinical tissue, and cell lines confirmed the expression of MKLN1-AS and TMCC1-AS1 were upregulated in LIHC and associated with a worse prognosis. The novel signature based on the two DRLs provide new insight into LIHC prognostic prediction, TME, and potential therapeutic strategies.

## Introduction

Liver cancer is one of the most prevalent malignancies globally, with an estimated 905,677 (4.7% cancer-related new cases) new cases and 830,180 (8.3% cancer-related death) deaths worldwide in 2020^1^. It is expected that there will be a 55.0% increase per year in new liver cancer cases between 2020 and 2040, with 1.4 million potential patients diagnosed in 2040^2^. Liver hepatocellular carcinoma (LIHC) is the dominant type of primary liver cancer, accounting for 85–90% of all cases^3^. The main risk factors for developing LIHC include chronic hepatitis B/C virus infection, alcoholism, aflatoxin exposure, and metabolic risk factors^4^. Despite great advances in early diagnosis with molecular markers and multiple therapies, 5-year survival rates for LIHC remain unsatisfactory^5^. Recurrence or metastasis occurs in approximately 70% of LIHC patients who undergo surgical resection^3^. Worse, LIHC is a heterogeneous disorder with widespread chemotherapy and radiation resistance. Therefore, it is critical to identify and validate novel prognostic and predictive biomarkers for LIHC, which may lead to efficient detection and discovery of optimal therapies for LIHC.

Regulatory cell death (RCD) is a common type of cell death that is characterized by uncontrolled cell expansion or accumulation^6^. RCD plays an essential role in cancer metabolic therapy^7^, tissue homeostasis, inflammation, and multiple pathophysiological conditions^6^. Over the past two decades, several emerging RCD modalities have been highlighted, such as cuproptosis^8^, ferroptosis^9^, apoptosis, necroptosis, pyroptosis, parthanatos, autophagy-dependent cell death, immunogenic cell death, and so on^6^. The physiological forms of RCD are generally regarded as key therapeutic targets for the management of multiple human diseases^6^. Most recently, Liu et al.^10^ provided the first insight into a novel form of RCD known as “disulfidptosis”, which differs from previously identified cell death modalities. This study revealed that excessive expression of solute carrier family 7 member 11 (SLC7A11) in glucose-deficient cancer cells accelerates nicotinamide adenine dinucleotide phosphate (NADPH) depletion under glucose starvation conditions^10^. This leads to an accumulation of disulfides that cannot be reduced, altering the conformation of cytoskeletal proteins and inducing disulfide stress, which ultimately leads to disulfidptosis, an unusual form of cell death with a specific underlying mechanism^7,10,11^. Disulfidptosis activation may require three elements: high SLC7A11 expression, glucose deprivation conditions that impair glucose metabolism, and aberrant disulfide bonds between actin cytoskeleton proteins^12^. Aberrant accumulation of intracellular disulfide molecules, such as lipoic acid and cystine, triggers disulfide stress and extremely rapid cell death^13^. Disulfidptosis is an unusual form of cell death in morphology, genetics, and biochemistry that is involved in the regulation of tumor progression^12^. A link between disulfidptosis and cancer has been established via bioinformatics analysis recently^11,14–18^. Yang et al. discovered disulfidptosis-based prognostic and tumor microenvironment (TME) characteristics of clear cell renal cell carcinoma and identified AJAP1 as a possible biomarker for the disease^11^. Zhao et al. investigated the role of disulfidptosis-related genes (DRGs) in bladder cancer prognosis and generated a risk signature to assess patient prognosis, TME, and immunotherapy^14^. Wang et al. developed a risk score for DRGs and discovered that SPP1 and MYBL2 genes might predict the survival of LIHC patients^15^. Currently, the role of disulfidptosis in the assessment of the occurrence, progression, prognosis, and treatment of LIHC is scarce. More indicators associated with disulfidptosis with cancer susceptibility are urgent to be established.

Long non-coding RNAs (lncRNAs) are RNA molecules length greater than 200 nucleotides that cannot code for protein^19^. Growing evidence suggests that the role of lncRNAs in basics, transcriptomic, and clinical oncology may be comparable to, and possibly greater than that of protein-coding genes^19^. Over the past decade, the research on lncRNA-based clinical tools has expanded rapidly, including in cancer diagnostic and prognostic biomarkers, and therapeutic targets^19^. LncRNA-related signatures for prognostic prediction of LIHC have attracted more and more attention, such as cuproptosis-related lncRNAs^20–22^, ferroptosis-related lncRNAs^23–25^, necroptosis-related lncRNAs^26,27^, pyroptosis-related lncRNAs^28–30^, and so on. However, disulfidptosis-related lncRNAs (DRLs) as prognostic markers for LIHC have never been investigated. Therefore, we aim to identify different clustering features and construct DRLs to predict the prognosis of LIHC patients, and evaluate the correlation between the signatures and the tumor microenvironment and drug sensitivity.

## Materials and methods

### Dataset and data acquisition

LIHC dataset was downloaded from the TCGA database (https://portal.gdc.cancer.gov/repository). The R (version 4.1.3, https://www.r-project.org/) and relative R packages were used to analyze the TCGA cohort. After obtaining from TCGA, an FPKM gene expression matrix was transformed into TPM format^31^. Next, using the R package “sva”, the merged expression matrix was normalized and eliminated from batch effects^32^.

### Identification of co-expressed DRGs and lncRNAs

Ten disulfidptosis-related genes (DRGs) were identified from the recently published studies^10,11^. Univariate cox regression method was applied to evaluate the hazard ratio of the DRGs to the LIHC. Then, the R package “limma” was used to acquire the expression of DRGs (see Supplementary Text files)^33^. According to previous documents, Pearson correlation analysis was performed to evaluate the relationship between DRGs and lncRNAs in the RNA-seq data of TCGA-LIHC samples. Next, to find adequate disulfidptosis-related lncRNAs (DRLs), we set correlation coefficient > 0.4 and P < 0.001 was the optimal cutoff value using the relevant R script (see Supplementary Text files)^22^. Moreover, the R packages “ggplot2” and “ggalluvial” were used to construct a Sankey diagram to show the correlation between DRGs and DRLs^34^.

### Construction of DRLs risk model

The LIHC patients were randomly assigned to the testing and training groups using the R package “caret” in a 1:1 ratio^35^. Univariate cox regression was utilized for obtaining prognostic DRLs (P < 0.01). Lasso cox regression analysis was used to construct the prediction model for DRLs^36,37^. Multivariate cox regression analysis was applied to build a two-lncRNA predictive model. The calculated formula used for disulfidptosis-related prognostic risk score was based on the published documents^22,38^. The model of risk score was calculated as follows: risk score = ∑i = LnCoef(i) × EXP(i). Patients were classified into low-risk and high-risk groups, depending on the median value of the risk score. The R package ‘survminer’ was used to construct Kaplan–Meier curves to examine the prognostic relevance of risk models for DRLs^39^. The R package “timeROC” was utilized to investigate the receiver operating characteristic (ROC) of survival^39^. In addition, univariate cox regression and multivariate cox regression methods were applied to evaluate the prognostic predictive potential of this risk score model. The P < 0.05 was selected by R software.

### Principal component analysis (PCA) analysis and clinical features

PCA analysis was performed using the R packages “scatterplot3d”^40^ to demonstrate whether the DRLs prognostic risk score model can identify low-risk and high-risk groups of patients to further clarify the clinical utility of DRLs. The association between this risk score model and clinical variables, such as grade, gender, age, and stage, were evaluated by chi-square tests and Wilcoxon rank-sum test.

### Nomogram and calibration curve

Using the DRLs risk model and clinicopathological factors (age, gender, grade, stage, tumor (T), metastasis (M), and positive lymph node (N)), a nomogram for predicting the 1/3/5-year survival of LIHC patients was created. We used a calibration curve to check if the nomogram predicted survival rate agreed with recognition survival rate. The R package “rms” and “regplot” were used to construct the nomogram^41^, and calibration curve^42^.

### Function enrichment analysis

For the purpose of investigating majorly enriched signaling pathways and biological roles involved in the disulfidptosis-related lncRNA signature, Gene Ontology (GO) and Kyoto Encyclopedia of Genes and Genomes (KEGG) were performed^43^. GO analysis was performed to identify the enriched pathways in different sets by R packages “clusterProfiler”, “org.Hs.eg.db”, “enrichplot”, “ggplot2”, “circlize”, “RColorBrewer”, “dplyr”, “ggpubr”, “ComplexHeatmap” with P = 0.05 and q = 0.05^44^. KEGG analysis was used to reveal the pathways associated with high- and low-risk sets caring out by using R packages “DOSE”, “clusterProfiler”, “org.Hs.eg.db”, “enrichplot”, “ggplot”, “circlize, “RColorBrewer”, “dplyr”, and “ComplexHeatmap” with P = 0.05 and q = 0.05^45–47^.

### Tumor microenvironment (TME) analysis

The proportion of tumor-infiltrating immune cells and immune function were displayed on boxplots for low- and high-risk sets created by R packages “limma”, “ggpubr”, and “reshape2”^33^. The level of immune cell infiltration in TME of LIHC was investigated using a single-sample gene set enrichment analysis (ssGSEA) algorithm^48^.

### Immune dysfunction and exclusion (TIDE) analysis

The TIDE scoring file was acquired from the website (http://tide.dfci.harvard.edu). The R package “ggpubr” was utilized to examine the difference in TIDE between the high- and low-risk sets^49^.

### Tumor mutation burden (TMB) analysis

The different analyses of TMB, survival analysis, the prognosis and tumor mutation between the high- and low-risk sets was performed using the R package “ggpubr” and “limma”^33^.

### Drug susceptibility analysis

We screened potential chemotherapeutic drugs using the R packages “pRRophetic”^50^ to determine whether high-risk and low-risk groups have significant differential susceptibilities to the therapy (IC50 value). The filtering threshold was set at P < 0.001.

### Clinical tissue samples

A total of 50 LIHC specimens and adjacent non-cancerous tissues were collected from LIHC patients who underwent surgical resection at the First Affiliated Hospital of Guangxi Medical University between June 2022 and March 2023. The following were the inclusion criteria: (I) pathologically diagnosed with LIHC the cancer for the primary time; (II) didn’t receive radiation, chemotherapy, targeted therapy, ablation, or intervention prior to the operation. Patients were excluded if they met any one of the following criteria: (I) the presence of other malignancies; (II) the occurrence of diabetes, hypertension, or other chronic conditions. All the tissues were stored at − 80 °C until the extraction of total RNA. Informed consent was obtained from all subjects involved in the study. The Medical Ethics Committee of First Affiliated Hospital of Guangxi Medical University has approved the protocol (No. 2023-E321-01).

### Cell lines

Normal LO2 cells and LIHC cells (Hep G2, Huh-7, Hep-3B, MHCC-97H, QGY-7703, SMMC-7721, and HCC-LM3) were bought from the Chinese Academy of Sciences Cell Bank in Shanghai, China. All cell lines were cultured in Dulbecco’s modified Eagle’s medium (Gibco, USA) supplemented with 10% fetal bovine serum (FBS, Gibco, USA), 100 U/mL streptomycin, and 100 U/mL penicillin in a 5% CO2 environment at 37 °C.

### RNA extraction and quantitative real-time polymerase chain reaction (qRT-PCR)

Total RNA from the tissues and cell lines was extracted using the TRIzol reagent. The quality and quantity of the RNA was then assessed using NanoDrop (Thermo Science). cDNA was synthesized using the Superscript II Reverse Transcriptase Kit (Takara, Japan). qRT-PCR was performed using the SYBR Green (Takara, Japan) method to detect transcript levels in tissues and cell lines by ABI 7500 Real-Time PCR System. The endogenous control gene glyceraldehyde 3-phosphate dehydrogenase (GAPDH) was used to normalize the expression of the target gene. The 2^−ΔΔT^ approach was used to calculate the relative expression value. Primer sequences for MKLN1-AS: (f) 5′-TGACCGACACTGGGTCTGA (r) 5′-AGGCTTTCAGGAGTCCAACC; TMCC1-AS1: (f) 5′-TGCCATGCCCGTGTCAACTG (r) 5′-CTCTCTCGTTCTGCCTCCCTTATG.

### Extended database validation

We used the GEPIA2: an updated and enhanced version of Gene Expression Profiling Interaction Analysis database (http://gepia2.cancer-pku.cn/#index)^51^ to further validate the credibility of the results. Gene expression, and overall survival (OS) survival analysis for MKLN1-AS and TMCC1-AS1 in LIHC samples were visually displayed. To divide high-expression and low-expression cohorts, the optimal cutoff was chosen automatically with log-rank P values.

### Statistical analysis

We used R software (version 4.1.3) and its associated packages to analyze all statistics. RNA-seq data, mutation data, clinical information, and Ensembl IDs for lncRNAs were extracted using Strawberry-Perl (version 5.30.0.1-64bit, https://www.perl.org). The transcript levels of MKLN1-AS and TMCC1-AS1 in LIHC tissues and para-noncancerous tissues were compared using a paired t-test. The transcript levels of MKLN1-AS and TMCC1-AS1 in normal LO2 cells and LIHC cells were compared using an unpaired t-test. The proportions of the clinical features were analyzed using the chi-squared test. Statistical significance was set at P < 0.05.

### Ethics approval and consent to participate

The study follows the principles of the Declaration of Helsinki. The study protocol was approved by the Ethics Committee of scientific research and clinical trial of the First Affiliated Hospital of Guangxi Medical University (Approval Number: 2023-E321-01). All patients provided written-informed consent for the collection and publication of their medical information at the first visit to our center, which was filed in their medical records.

## Results

### Identification and construction of DRLs risk model

**Figure 1:**
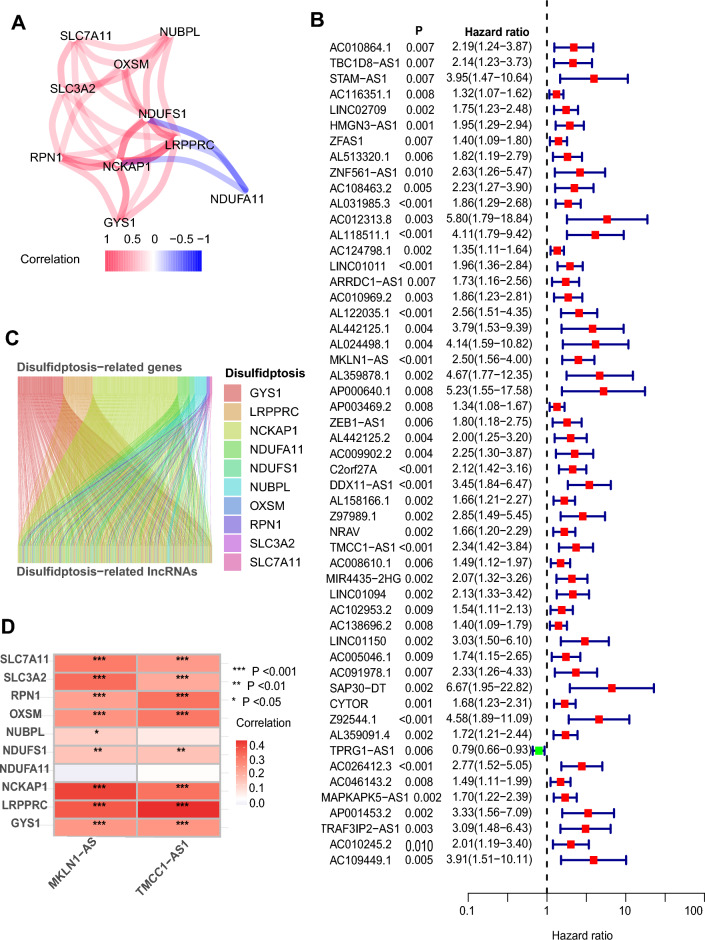
Construction of the differentially expressed disulfidptosis-related lncRNAs (DRLs) in TCGA-LIHC cohort. (**A**) The ten disulfidptosis-related genes (DRGs) and their correlation. (**B**) The significant prognostic DRLs were identified using univariate regression analysis. (**C**) The Sankey relation between DRGs and DRLs. (**D**) Heatmap of the correlation between DRGs and DRLs involved in model construction (*P < 0.05, **P < 0.01, ***P < 0.001, ****P < 0.0001).

RNA-seq data of 424 samples (374 tumor and 50 normal tissues) were downloaded from TCGA database. Individuals with incomplete genomes or clinical data were eliminated (n = 4), leaving 370 LIHC samples in the final group. In recent literature^10^, authors proposed a unique form of cell death termed disulfidptosis, which was not characterized previously. Ten DRGs were obtained from studies, and their details and correlation were presented in Fig. 1A and Supplementary Table S1. Then, a total of 1354 DRLs were obtained by Pearson analysis on the basis of R > 0.4 and P < 0.001. The significant prognostic DRLs were identified using univariate regression analysis (Fig. 1B) and Lasso cox regression analysis (see Supplementary Fig. S1A,B). The correlation between DRGs and DRLs was displayed in Fig. 1C and Supplementary Table S2. Finally, two key candidate OS-related DRLs were extracted to construct the prognostic signature, which include MKLN1-AS and TMCC1-AS1. The relation between the two DRLs and the DRGs was shown in Fig. 1D.

The expression of these 10 genes between tumor and normal tissues in LIHC patients was showed in Supplementary Fig. S1C. The hazard ratio of the ten genes on the prognosis in LIHC patients was showed in Supplementary Fig. S1D. A total of 370 LIHC patients were randomly divided into the training set (n = 185) and the testing set (n = 185). Table 1 showed the clinical characteristics of the two groups of patients, demonstrating that there was no variation in clinical characteristics between the training and testing groups.

**Table 1:** Clinical characteristics of 370 LIHC patients stratified by test and train sets.

Covariates	Type	Total	Test	Train	P
Age	≤ 65	232 (62.7%)	125 (67.57%)	107 (57.84%)	0.068
	> 65	138 (37.3%)	60 (32.43%)	78 (42.16%)	
Gender	Female	121 (32.7%)	60 (32.43%)	61 (32.97%)	1.00
	Male	249 (67.3%)	125 (67.57%)	124 (67.03%)	
Grade	G1	55 (14.86%)	34 (18.38%)	21 (11.35%)	0.195
	G2	177 (47.84%)	82 (44.32%)	95 (51.35%)	
	G3	121 (32.7%)	64 (34.59%)	57 (30.81%)	
	G4	12 (3.24%)	5 (2.7%)	7 (3.78%)	
	Unknown	5 (1.35%)	0 (0%)	5 (2.7%)	
Stage	Stage I	171 (46.22%)	89 (48.11%)	82 (44.32%)	0.640
	Stage II	85 (22.97%)	38 (20.54%)	47 (25.41%)	
	Stage III	85 (22.97%)	45 (24.32%)	40 (21.62%)	
	Stage IV	5 (1.35%)	3 (1.62%)	2 (1.08%)	
	Unknown	24 (6.49%)	10 (5.41%)	14 (7.57%)	
Tumor (T)	T1	181 (48.92%)	92 (49.73%)	89 (48.11%)	0.721
	T2	91 (24.59%)	42 (22.7%)	49 (26.49%)	
	T2a	1 (0.27%)	1 (0.54%)	0 (0%)	
	T2b	1 (0.27%)	0 (0%)	1 (0.54%)	
	T3	80 (21.62%)	42 (22.7%)	38 (20.54%)	
	T4	13 (3.51%)	7 (3.78%)	6 (3.24%)	
	Unknown	3 (0.81%)	1 (0.54%)	2 (1.08%)	
Metastasis (M)	M0	266 (71.89%)	139 (75.14%)	127 (68.65%)	0.689
	M1	4 (1.08%)	3 (1.62%)	1 (0.54%)	
	Unknown	100 (27.03%)	43 (23.24%)	57 (30.81%)	
Lymph node (N)	N0	252 (68.11%)	129 (69.73%)	123 (66.49%)	1.00
	N1	4 (1.08%)	2 (1.08%)	2 (1.08%)	
	Unknown	114 (30.81%)	54 (29.19%)	60 (32.43%)	

### Predicting the prognosis of LIHC patients with the DRLs predictive model

**Figure 2:**
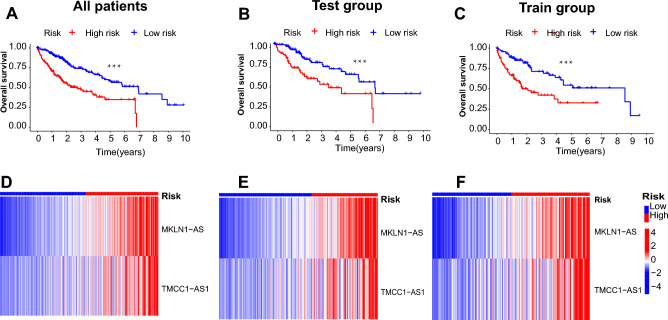
Construction and evaluating of the prognostic value of disulfidptosis-related lncRNAs (DRLs) in all LIHC patients, test, and train sets. (**A–C**) Kaplan–Meier (KM) survival analysis between high-risk and low-risk LIHC patients. (**D–F**) Heatmaps of two DRLs between high-risk and low-risk LIHC patients (*P < 0.05, **P < 0.01, ***P < 0.001, ****P < 0.0001).

The coefficients of the two DRLs were used to assess the scores for each patient. The risk score was calculated as follows: risk score = expression of MKLN1-AS × 1.010769 + expression of TMCC1-AS1 × 0.692979. Next, LIHC patients were divided into high-risk and low-risk groups based on the median risk score value (Supplementary Fig. S2A–C). Patients exhibited a higher mortality rate with risk scores increasing (Supplementary Fig. S2D–F). Survival analysis revealed that high-risk patients had significantly worse overall survival than low-risk patients (Fig. 2A–C; all P < 0.001). The expression of MKLN1-AS and TMCC1-AS1 was higher in the high-risk patients than in the low-risk patients in all sets (Fig. 2D–F), indicating that the two DRLs might be the poor indicators of prognosis.

**Figure 3:**
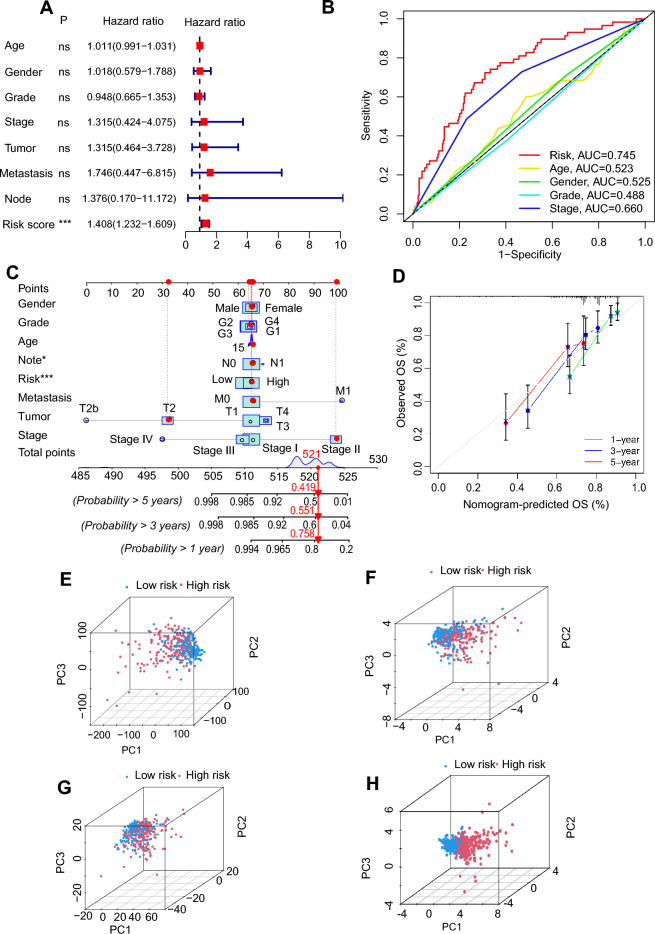
Validation of the prognostic value of the disulfidptosis-related lncRNA (DRLs) signature model. (**A**) Multivariate cox regression analysis of the clinical variables and DRLs risk scores model. (**B**) Comparison of predictive risk score model and clinicopathological features. (**C**) A nomogram showing risk and clinicopathological parameters for predicting 1-, 3- and 5-OS in LIHC patients. (**D**) Calibration curves indicating the accuracy of risk model to predict 1-, 3- and 5-OS of LIHC patients. (**E–H**) PCA analysis of DRLs and clinical parameters in LIHC patients: (**E**) PCA of all genes. (**F**) PCA of disulfidptosis-associated genes. (**G**) PCA of disulfidptosis-associated lncRNAs. (**H**) PCA of the risk model (*P < 0.05, **P < 0.01, ***P < 0.001 ****P < 0.0001, *ns* no significance).

Univariate cox regression (see Supplementary Fig. S3A) and multivariate cox regression (Fig. 3A) analysis revealed that risk score was an independent prognostic factor for LIHC patients. The risk score’s area under the curve (AUC) was 0.745 (Fig. 3B), greater than that of the other clinical parameters, indicating that it was a more accurate predictor of LIHC patients’ overall survival. Furthermore, the survival probability of 1-, 3- and 5-year for the LIHC patient in the nomogram was 0.758, 0.551, and 0.419, respectively (Fig. 3C). The nomogram predictions were validated by the calibration curves showing there was a high degree of congruence between the clinical outcomes and predictive overall survival rates at 1, 3, and 5 years (Fig. 3D). The risk score showed its value in evaluating the 1-, 3- and 5-year survival of patients (see Supplementary Fig. S3B). In addition, ROC curves showed that the 10-year concordance-index (C-index) scored higher in the risk model than other clinical variables (see Supplementary Fig. S3C). The survival probability and clinical parameters of LIHC patients were compared according to the gender, grade, stage, and tumor between the two risk groups (see Supplementary Fig. S3D–I). The results demonstrated that all clinicopathological variables were significant differences between the high- and low-risk groups, suggesting that patients with high-risk scores had a poor prognosis (all P < 0.05).

### PCA analysis

PCA analysis was performed on the models of all genes, DRGs, DRLs, and risk lncRNAs model (Fig. 3E–H). The results indicated that our constructed model was all able to effectively classify high-risk and low-risk LIHC patients, demonstrating the accuracy of the model. All of the above findings suggest that risk scores could be a reliable prognostic factor in predicting survival in LIHC patients.

### Functional and pathway analysis

**Figure 4:**
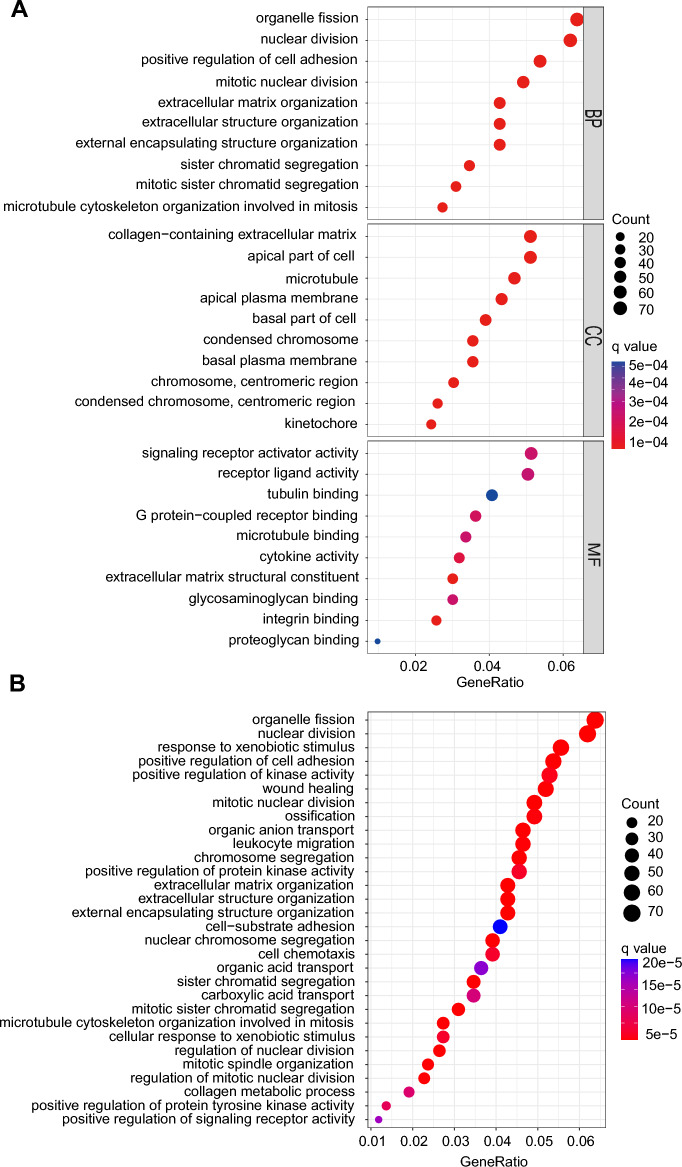
Gene Ontology (GO) and Kyoto Encyclopedia of Genes and Genomes (KEGG) pathway enrichment analyses between high- and low-risk groups. (**A**) GO analysis revealed the diversity of molecular biological process (BP), cellular component (CC) and molecular function (MF). (**B**) KEGG pathway analysis revealed the significantly enriched pathways.

GO and KEGG analyses were used to investigate the underlying different biological properties of genes in high-risk and low-risk subsets. A total of 1405 differently expressed genes were discovered between the high-risk and low-risk groups. (Supplementary Table S3). According to GO pathway analysis (Fig. 4A), biological process (BP) terminology indicated that differently expressed genes are rich in the “organelle fission”, “nuclear division”, and “positive regulation of cell adhesion”. Cell composition (CC) showed differently expressed genes were mainly enriched in “collagen-containing extracellular matrix” “apical part of cell” and “microtubule”. In term of molecular function (MF), “signaling receptor activator activity”, “receptor ligand activity” and “tubulin binding” were significantly abundant. KEGG pathway analysis (Fig. 4B) indicated that differently expressed genes were mainly concentrated in “organelle fission”, “nuclear division”, and “positive regulation of cell adhesion”. According to the results, we proposed that the DRGs primarily affect the cellular metabolic activities.

### Tumor microenvironment (TME) analysis

**Figure 5:**
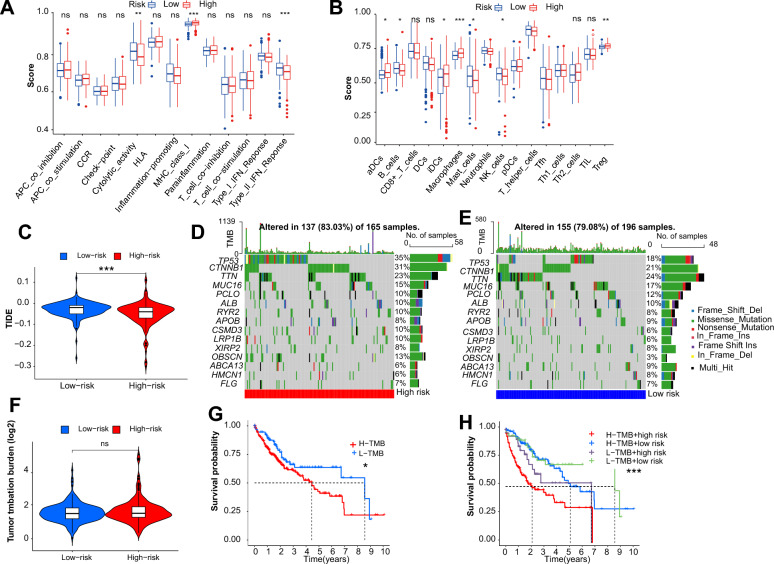
Tumor microenvironment (TME), immune dysfunction and exclusion (TIDE), and tumor mutation burden (TMB) analysis of the risk score between the low and high-risk groups. (**A**) Immune function differences analysis. (**B**) Tumor-infiltrating immune cells differences analysis. (**C**) TIDE analysis. (**D,E**) The waterfall plot showed the mutated genes. (**F**) TMB analysis of the risk score. (**G,H**) TMB survival analysis and risk score (*P < 0.05, **P < 0.01, ***P < 0.001, ****P < 0.0001, *ns* no significance).

The correlation of immune function differences between the low- and high-risk groups was depicted in Fig. 5A. “Cytolytic_activity”, “MHC_class_I”, and “Type_II_IFN_Reponse” were significantly different between the low- and high-risk groups. Furthermore, the relationship between the risk score and the content of tumor-infiltrating immune cells were surveyed. As illustrated in Fig. 5B, the “aDCs”, “B_cells”, “iDCs”, “Macrophages”, “Mast_cells”, “NK_cells”, and “Treg” were substantially different between high-risk and low-risk groups. The high-risk group had significantly higher proportion of “aDCs”, “Macrophages” and “Treg” whereas the low-risk group exhibited greater percentage of “B cells,” “Mast_cells”, and “NK_cells”. These results indicated that disulfidptosis acts a vital role in the formation of the TME.

### Immune dysfunction and exclusion (TIDE) analysis of the risk score

Immune escape and immunotherapy differences between high and low risk groups were examined to determine the impact of immunotherapy in LIHC patient populations. The clinical response to immune checkpoint blocking (ICB) therapy was evaluated using the TIDE score. A high TIDE score implies a low response to ICB and a poor prognosis for the cancer. TIDE score was lower in the high-risk group (Fig. 5C; P < 0.001). The lower composite TIDE score attained in the high-risk group could be explained with a higher proportion of treatment remissions.

### Tumor mutation burden (TMB) analysis of the risk score

Using the “maftools” software package, we examined the somatic mutation scope of LIHC patients in high- and low-risk groups and chose 15 genes (TP53, CTNNB1, TTN, MUC16, PCLO, ALB, RYR2, APOB, CSMD3, LRP1B, XIRP2, OBSCN, ABCA13, HMCN1, FLG) with the highest mutation frequency for representation (Fig. 5D,E). The waterfall plot revealed that the top three mutated genes in high risk LIHC samples were TP53 (35%), CTNNB1 (31%), and TTN (23%), whereas in low risk LIHC samples, TTN (24%), CTNNB1 (21%), and TP53 (18%) were the most common alteration genes. In all groups, the most common variation categorization was missense mutation. Overall, there were no significant differences in TMB between the low- and high-risk groups (Fig. 5F; P = 0.42). According to a TMB survival analysis, patients in the high TMB group had a poorer prognosis (Fig. 5G; P = 0.031). When TMB and patient risk scores combined, the survival rate of high TMB + high risk score groups had the poorest prognosis (Fig. 5H; P < 0.001).

### Drug susceptibility analysis

**Figure 6:**
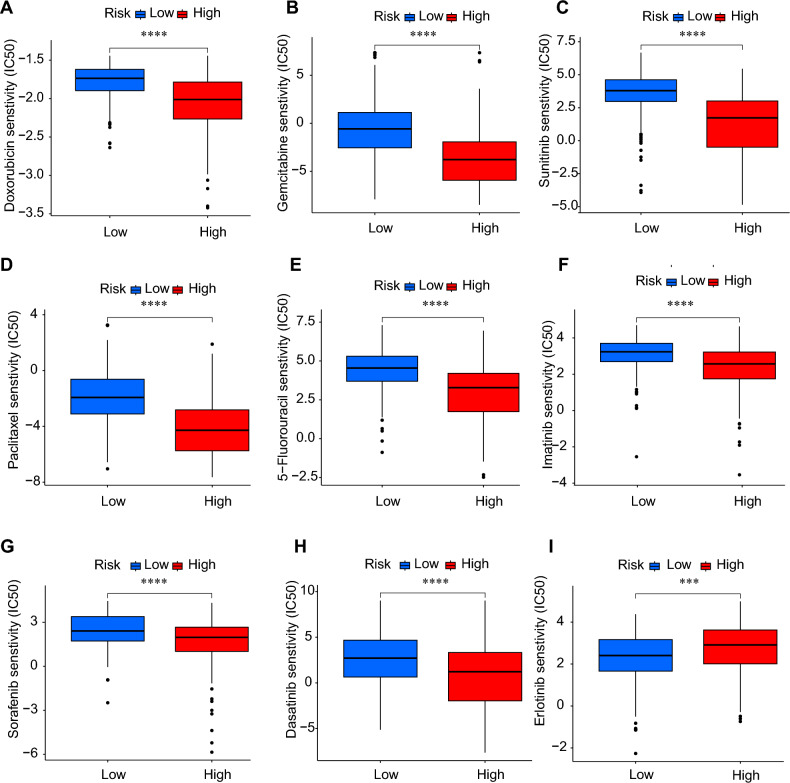
Drug sensitivity analysis and screening potential drugs for LIHC between two groups. (**A**) Doxorubicin; (**B**) gemcitabine; (**C**) sunitinib; (**D**) paclitaxel; (**E**) 5-fluorouracil; (**F**) imatinib, (**G**) sorafenib, (**H**) dasatinib; (**I**) erlotinib (*P < 0.05, **P < 0.01, ***P < 0.001 ****P < 0.0001).

To determine whether the high-risk and low-risk groups had significantly different susceptibilities to the chemotherapy, we performed drug sensitivity analysis using “pRRophetic” package to screen prospective anti-tumor drugs (IC50 value). The sensitivity to medicines increases with decreasing IC50 values. Because too many chemotherapeutic drugs were screened based on the two DRLs, we picked nine chemotherapy agents with great differences including sorafenib, 5-fluorouracil, dasatinib, doxorubicin, imatinib, gemcitabine, sunitinib, paclitaxel, and erlotinib commonly used in clinical settings to do our further research. Doxorubicin (Fig. 6A), gemcitabine (Fig. 6B), sunitinib (Fig. 6C), paclitaxel (Fig. 6D), 5-fluorouracil (Fig. 6E), imatinib (Fig. 6F), sorafenib (Fig. 6G), and dasatinib (Fig. 6H) are eight of the nine treatments with better sensitivity/lower IC50 in the high-risk group, suggesting that these eight medications are more likely to be beneficial for high-risk LIHC patients (P < 0.0001). Erlotinib (Fig. 6I) is only one of nine treatments with better sensitivity/lower IC50 in the low-risk group (P < 0.001).

### Validation data

**Figure 7:**
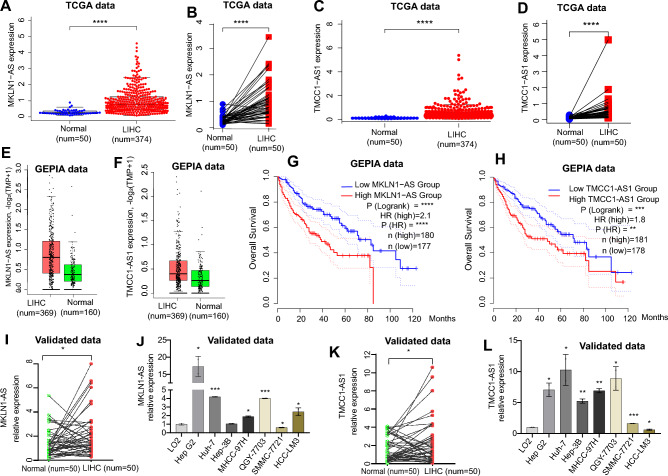
The relative expression levels of two candidate lncRNAs in LIHC tissues compared with normal tissues, cell lines, and validation dataset. The expression of MKLN1-AS (**A,B**) and TMCC1-AS1 (**C,D**) in 374 LIHC tissues compared with 50 normal tissues in the TCGA database. The expression of MKLN1-AS (**E**) and TMCC1-AS1 (**F**) in 369 LIHC tissues compared with 160 normal tissues in the GEPAI database. Overall survival (OS) of different MKLN1-AS (**G**) and TMCC1-AS1 (**H**) expression level in LIHC samples. The expression of MKLN1-AS (**I**) and TMCC1-AS1 (**K**) in 50 pairs of LIHC and adjacent normal tissues in the validation dataset. The expression of MKLN1-AS (**J**) and TMCC1-AS1 (**L**) in HCC cell lines (*P < 0.05, **P < 0.01, ***P < 0.001, ****P < 0.0001).

The relative expression levels of MKLN1-AS and TMCC1-AS1 in LIHC tissues and adjacent normal tissues, cell lines, and validation dataset were showed in Fig. 7. According to the TCGA and GEPIA2 data, the expression levels of these two lncRNAs were both considerably increased in LIHC tissues when compared to nearby normal tissues (Fig. 7A–F). MKLN1-AS and TMCC1-AS1 transcription levels were also found to be strongly linked with a poor prognosis of LIHC (Fig. 7G,H). We used qRT-PCR to examine the expression levels of MKLN1-AS and TMCC1-AS1 in 50 pairs of LIHC tissue and adjacent normal tissue (see Supplementary Original data). MKLN1-AS and TMCC1-AS1 transcription levels were significantly higher in LIHC tissues compared to adjacent normal tissues, which was consistent with the TCGA and GEPIA2 database results (Fig. 7I,K). The expression of the two lncRNAs in LIHC cells was compared to that of normal LO2 cells and LIHC cells (Hep G2, Huh-7, Hep-3B, MHCC-97H, QGY-7703, SMMC-7721, and HCC-LM3) (see Supplementary Original data). MKLN1-AS was considerably higher in Hep G2, Huh-7, MHCC-97H, QGY-7703, and HCC-LM3 cells than in normal LO2 cells (Fig. 7J), while TMCC1-AS1 was significantly higher in Hep G2, Huh-7, Hep-3B, MHCC-97H, QGY-7703,and SMMC-7721 cells (Fig. 7L). Overall, the results demonstrated that the MKLN1-AS and TMCC1-AS1 expression levels were up-regulated in LIHC and associated with the worse prognosis of LIHC.

## Discussion

RCD plays a crucial role in regulating tumor proliferation and cell metabolism^6^. Disulfidptosis, a novel metabolic-related RCD proposed in 2023, was suggested to provide a therapeutic strategy to target disulfidptosis in cancer therapy^10^. Currently, there is a dearth of research on the role of disulfidptosis in the oncogenesis, progression, prognosis, immune profile, signaling pathways, and targeted therapy for LIHC. In the present study, 374 LIHC samples and 50 normal controls were obtained from the TCGA database. We focused on the expression levels of DRLs (MKLN1-AS and TMCC1-AS1) and the clinical features of LIHC to construct a comprehensive disulfidptosis-related predictive model. Based on the expression levels of the DRLs, the LIHC sample was divided into low-risk and high-risk group to investigate the effect of this disulfidptosis pattern on LIHC prognosis and immunotherapy response. In addition, 369 LIHC samples and 160 normal controls from the GEPIA2 database were used to further validate the credibility of the results. We also used qRT-PCR to examine the expression levels of MKLN1-AS and TMCC1-AS1 in 50 pairs of LIHC tissue and adjacent normal tissue, as well as in LIHC cell lines. Our data demonstrated that MKLN1-AS and TMCC1-AS1 expression levels were upregulated in LIHC and associated with a worse prognosis. In addition, the LIHC patient in high-risk group was associated with poor OS. GO and KEGG data demonstrated that DRGs primarily affect the cellular metabolic activities. Moreover, the tumor-infiltrating immune cells were significantly different between the high-risk and low-risk groups. Surprisingly, the TIDE results showed a higher immune escape potential in the low-risk group, suggesting that immunotherapy was less effective in low-risk LIHC patients. Finally, LIHC patients in the high-risk group were more sensitive to several potential chemotherapeutic drugs. Taken together, the novel signature based on the two DRLs provide new insight into LIHC prognostic prediction, immune microenvironment characteristics, and potential therapeutic strategies using a combination of disulfidptosis and drug sensitivity.

In this study, we identified ten genes that suppress disulfidptosis based on recent publications^10,11^. Six of these ten DRGs, namely GYS1, LRPPRC, NCKAPA1, RPN1, SLC3A2, and SLC7A11, were significantly associated with the risk of LIHC. It is well established that SLC7A11 is overexpressed in multiple human cancers and protects cancer cells from amino acid deprivation, oxidative stress, and metabolic stress^52^. Mechanistically, high levels of SLC7A11 expression, H_2_O_2_ therapy, and cystine uptake in tumor cells trigger the accumulation of harmful disulfide molecules and NADPH depletion, eventually resulting in rapid cell death—disulfidptosis^12,53^. Currently, few research has been conducted to investigate the involvement of DRGs in cancer^11,14–17^. Most recently, Feng et al. used 24 DRGs to create a classification and model that can effectively predict the prognosis and drug sensitivity of thyroid carcinoma patients^17^. Besides, Chen et al. reported a DRG prognostic model with ten features and found that bladder cancer patients with high DRG scores may have worse survival, inflamed TME, and an increased TMB, suggesting that the model could be used for personalized therapy^16^.

Increasing evidence suggests that the tumor microenvironment (TME) plays a crucial role in the progression and metastasis of tumors^54^. The TME of LIHC comprises extracellular matrix, endothelial cells, stromal cells, as well as various cytokines and proteins^55^. The infiltration of multiple immune cells within the TME promotes tumor cell growth, immune tolerance, and immune evasion, thereby facilitating cancer cell metastasis^54^. In this study, we observed a significant correlation between MHC_class_I responses and immune function enrichment specifically in the high-risk group. Furthermore, it has been observed that the high-risk cohort exhibited a significantly increased proportion of antigen-presenting dendritic cells (aDCs), macrophages, and regulatory T cells (Treg cells). Importantly, tumor-infiltrating macrophages have been identified as potent producers of diverse mediators within the tumor microenvironment (TME), thereby facilitating tumor proliferation, metastasis, and invasion, ultimately leading to an unfavorable prognosis^56,57^. Tregs play a crucial role in maintaining self-tolerance within the immune system, suppressing antitumor immunity, facilitating tumor invasion and migration, and are closely associated with cancer development and progression^58^. The presence of Tregs infiltrating tumors has been consistently linked to unfavorable survival outcomes in various malignancies, including LIHC^58^. One study revealed that the high subtypes of disulfidptosis-related gene exhibited elevated levels of immune cells and immune scores in LIHC^15^. Furthermore, Yang et al. demonstrated a significant increase in the infiltration level of immune cells within the high disulfidptosis-related gene score group^59^. Additionally, there was a positive correlation between high disulfidptosis-related gene (SLC7A11) expression in LIHC and T helper cells, macrophages, and NK CD56 bright cells^60^. Our study consistently demonstrated that patients with LIHC in the high-risk group exhibited higher levels of activated dendritic cells (aDCs), macrophages, and Tregs compared to those in the low-risk group. The role of B cells in LIHC proliferation and metastasis remains unclarified based on recent studies^54^. On one hand, research has suggested that the loss of specific TGF-β on the surface of B cells could inhibit LIHC development^61^. On the other hand, a study discovered that CXCR3+ B cells could interact with the LIHC microenvironment to promote polarization of M2b macrophages and enhance metastatic potential^62^. In this study, we observed that the low-risk group with a better prognosis had a higher percentage of B cells.

Emerging research highlights critical roles of lncRNAs to the LIHC development and progression^63–65^. Recently, Chi et al. found that the lncRNA PTOV1-AS1 is up-regulation in LIHC and associated with patients’ prognosis and sorafenib resistance^64^. Another study revealed that LINC01977 accelerated LIHC progression by inhibiting Notch2 ubiquitination and depletion, suggesting that LINC01977 could be a potential biomarker and therapeutic target for LIHC patients^63^. Similarly, Sun et al. indicated that LINC01124 played a tumor-promoting role in LIHC through regulating the miR-1247-5p-FOXO3 axis^65^. Interestingly, the crucial role of RCD-related abnormally expressed lncRNAs in monitoring and promoting the development and progression of LIHC is drawing growing interest. Several RCD related lncRNA signatures to forecast prognosis and tumor progression in LIHC patients have been established, such as cuproptosis-related lncRNAs^20–22^, ferroptosis-related lncRNAs^23–25^, necroptosis-related lncRNAs^26,27^, pyroptosis-related lncRNAs^28–30^, and so on. For instance, Guo et al. established a cuproptosis-prognostic signature for four cuproptosis-related lncRNAs (SNHG4, AC026412.3, AL590705.3, and CDKN2A-DT) that could predict prognosis and evaluate the efficacy of immunotherapy for LIHC^20^. Liu et al. constructed a predictive model based on eight cuproptosis-related lncRNAs (AC004112.1, AC007064.2, AC012186.2, AC026412.3, AL031985.3, AL133477.1, AL365361.1, and TMCC1-AS1) to give valuable data for predicting the prognosis of LIHC patients and developing individualized targeted therapy^21^. Li et al. built a five cuproptosis-related lncRNAs (FOXD2-AS1, NRAV, MED8-AS1, WARS2-AS1, and MKLN1-AS) predictive signature for LIHC and confirmed the results of bioinformatics analysis using LIHC tissue samples and cell lines^22^. Li et al. concluded that the predictive signature could independently predict the prognosis of LIHC patients and provide treatment references for LIHC patients^22^. However, the role of DRLs in the development and progression of LIHC has never been investigated. Therefore, we estimated a risk signature based on DRLs to predict the survival of LIHC patients. Ten DRGs and 1354 DRLs were co-expressed from the transcriptome data of 374 LIHC samples from the TCGA database. Among them, 160 DRLs were significantly associated with prognosis of the LIHC patients. Subsequently, two DRLs (MKLN1-AS and TMCC1-AS1) were identified by LASSO and multivariate cox regression analysis. This is the first investigation of the role of lncRNA signatures associated with disulfidptosis in the prediction of LIHC prognosis and immune landscape, as well as drug sensitivity. Previously, MKLN1-AS and TMCC1-AS1 were identified as lncRNAs related to cuproptosis^22,66^, pyroptosis^67^, autophagy^68^, ferroptosis and necroptosis^27,69^ in LIHC. For example, Zhang et al. used lncRNA expression data from TCGA to establish that five necroptosis-related lncRNAs (KDM4A-AS1, ZFPM2-AS1, AC099850.3, MKLN1-AS, and BACE1-AS) were well associated with patient prognosis, clinicopathological features, and immunotherapy effects^69^. More recently, a six-lncRNA prognosis model (AC012073.1, AL031985.3, LINC01060, MKLN1-AS, MSC-AS1, and TMCC1-AS1) was construed by Zhu et al.^70^, and this was with the potential prognosis and immunotherapy response predictive value of LIHC. On the other hand, the functional role of MKLN1AS or TMCC1‑AS1 in LIHC progression has also been reported. Gao et al. found that MKLN1AS promoted LIHC progression by acting as a molecular sponge for miR6543p to promote hepatoma‑derived growth factor (HDGF) expression^71^. The researchers concluded that the MKLN1AS/miR654/3p/HDGF axis might serve as a potential target for LIHC diagnosis, prognosis, and therapy^71^. Another study revealed that MKLN1-AS overexpression contributes to stabilizing Yes-associated transcriptional regulator 1 (YAP1) mRNA, which is essential for MKLN1-AS to be carcinogenic^72^. Chen et al. reported that TMCC1-AS1 were significantly overexpressed in LIHC tissues and cell lines and promoted the proliferation, migration, invasion, suggesting TMCC1-AS1 could serve as a prognostic biomarker for LIHC patients^73^. Collectively, both of these critical lncRNAs have a role in LIHC development and progression. According to the findings of Liu et al.^10^, high expression of SLC7A11 in kidney cancer cells speeds up the depletion of NADPH in the cytoplasm under glucose starvation. This leads to an accumulation of unreducible disulfides, inducing disulfide stress and eventually disulfidptosis. As one of the DRGs, SLC7A11 upregulating plays an essential role in promoting disulfidptosis and tumor development^7,74^, which is harmful for cancer patients. This observation implies a positive relationship between DRGs and the two target lncRNAs. This was the first study to demonstrate that these two lncRNAs were identified to be involved in the development of LIHC disulfidptosis.

There are some limitations in this study. First, our results were obtained from the TCGA database, and we were unable to extract external validation from the GEO database due to the lack of relevant clinical data for the two lncRNAs. Second, the findings of our research depended mostly on integrative bioinformatics and preliminary qRT-PCR results. More basic research is needed to confirm the biological function of our identified lncRNAs in disulfidptosis.

In conclusion, we succeeded in firstly constructing a signature of disulfidptosis-related lncRNAs that predicts prognosis of LIHC patients. Besides, this signature can be a valuable tool for a deeper understanding of tumor immune microenvironment and the immunotherapy response of LIHC, providing us new insights into the therapeutic strategies of LIHC patients.

### Supplementary Information

Supplementary Figures.

Supplementary Information 1.

Supplementary Tables.

Supplementary Information 2.

## Data Availability

The datasets used and/or analyzed during the current study are available from the corresponding author on reasonable request.
