# Supplementary material for: A disulfidptosis-related lncRNAs signature in hepatocellular carcinoma: prognostic prediction, tumor immune microenvironment and drug susceptibility
Source: Sci Rep. 2024 Jan 7;14:746. doi: 10.1038/s41598-024-51459-z (PMC10772085; doi:10.1038/s41598-024-51459-z)
Supplement: Supplementary file 1 — Supplementary Figures. [file 41598_2024_51459_MOESM1_ESM.docx]

**Supplementary Figures**

Figure S1

A B


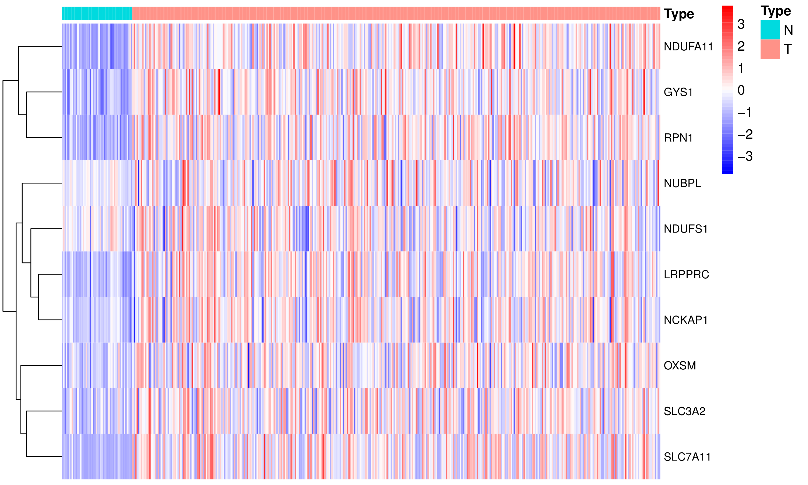

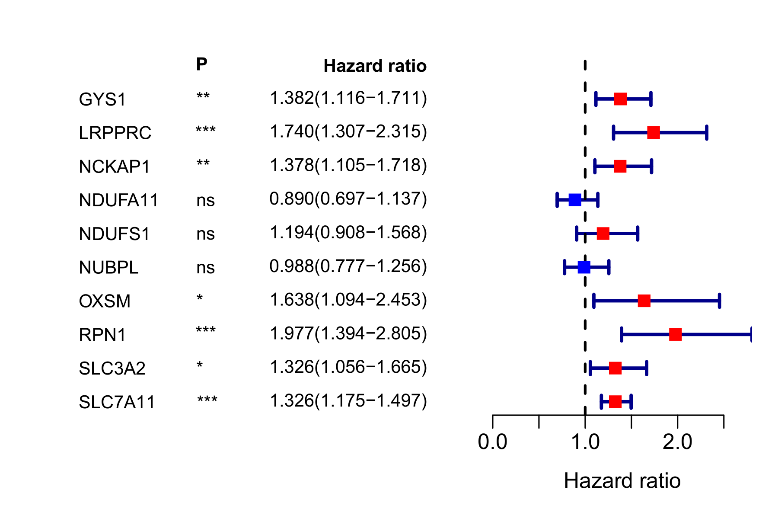


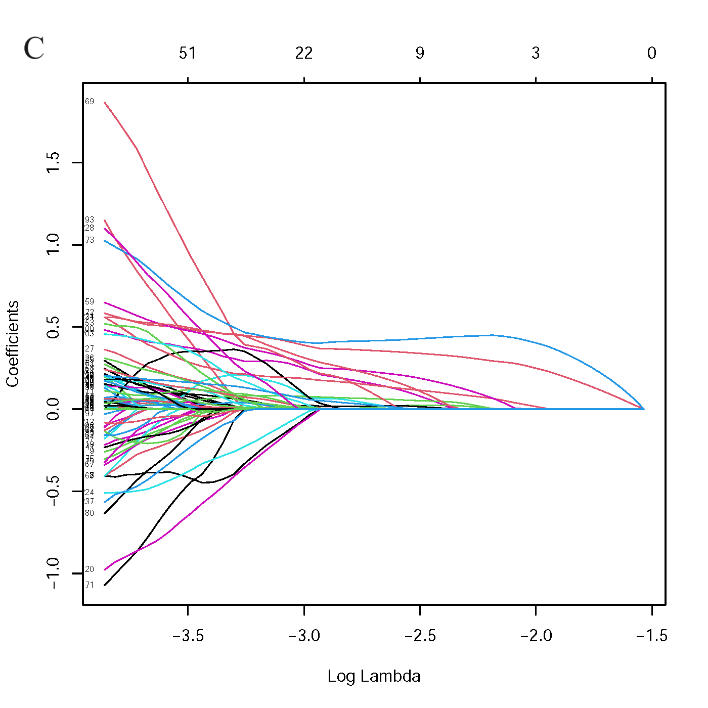

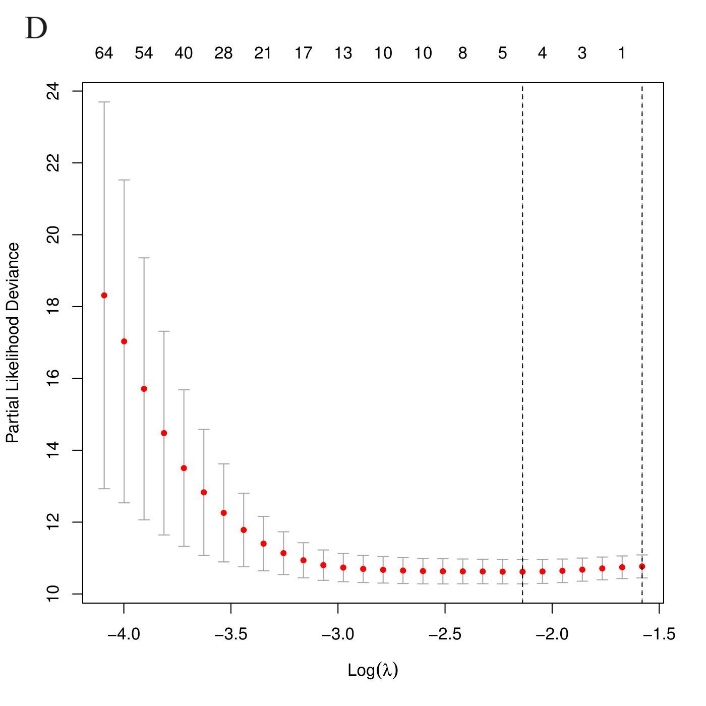


**Figure S1. Construction of the differentially expressed disulfidptosis-related lncRNAs (DRLs) in TCGA-liver hepatocellular carcinoma (HCC) cohort.** (A) Heatmap of expression of disulfidptosis-related genes (DRGs) in normal and LIHC tissue. (B) Hazard ratio of the DRGs to the LIHC. (C, D) The coefficient profile of prognostic genes by Lasso regression analysis. (*P < 0.05, **P< 0.01, ***P < 0.001 ****P<0.0001, ns: no significance)


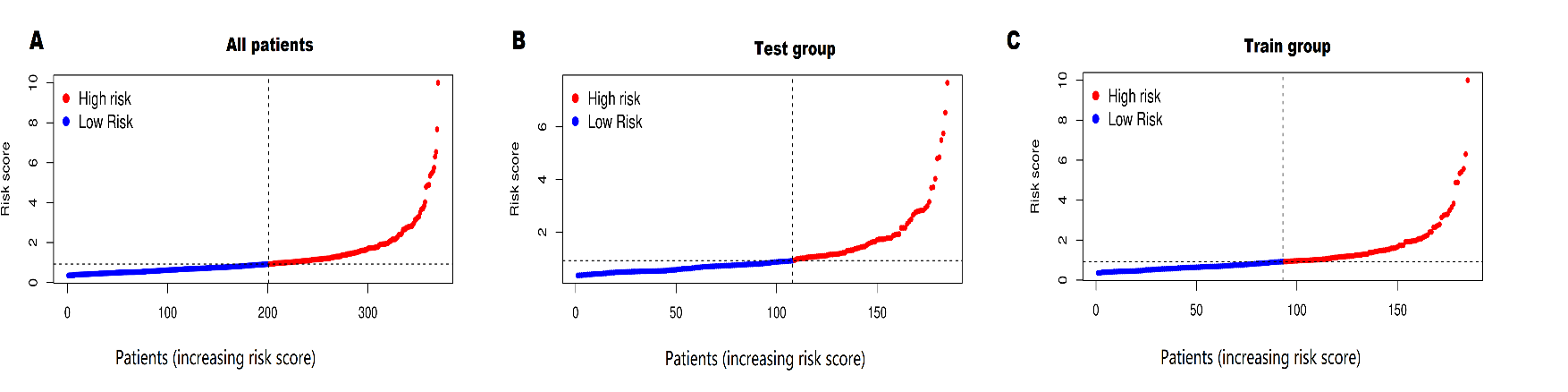
Figure S2


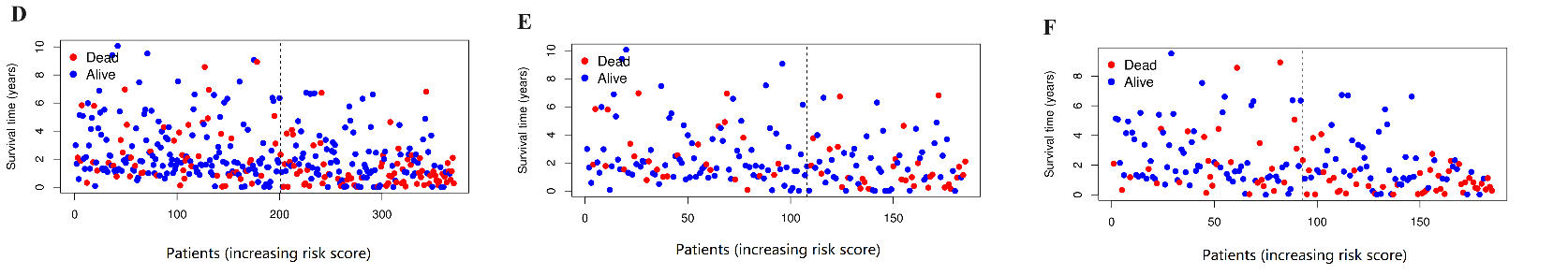


**Figure S2. Construction and evaluating of the prognostic value of disulfidptosis-related lncRNAs (DRLs) in all LIHC patients, test, and train sets.** (A-C) LIHC patients were divided into high-risk and low-risk groups based on the median risk score value. (D-F) Survival time of LIHC patients and risk scores.

Figure S3


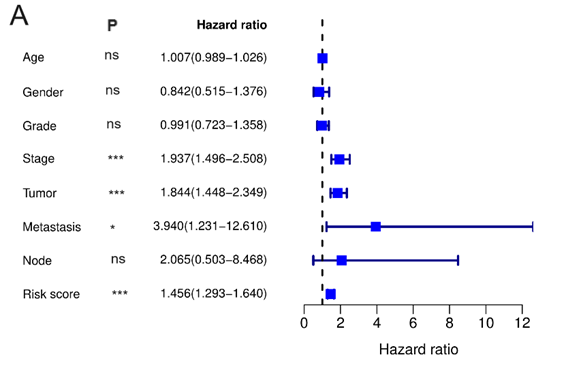

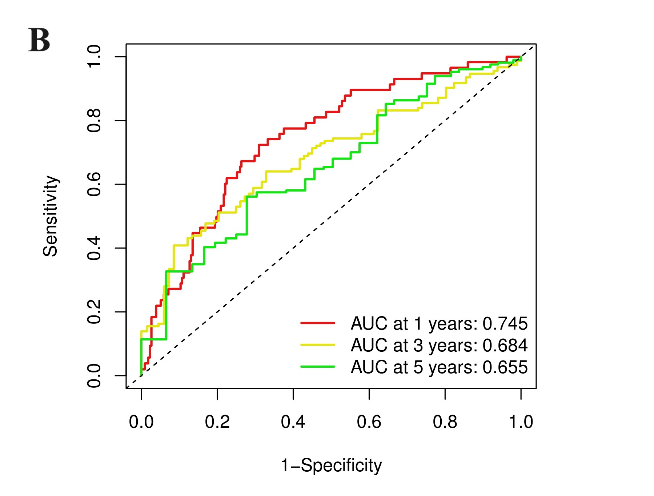

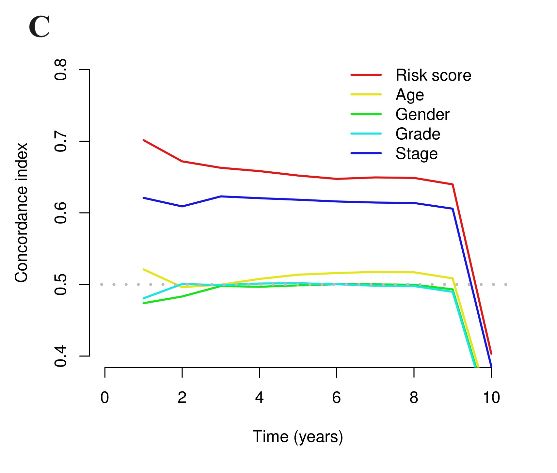

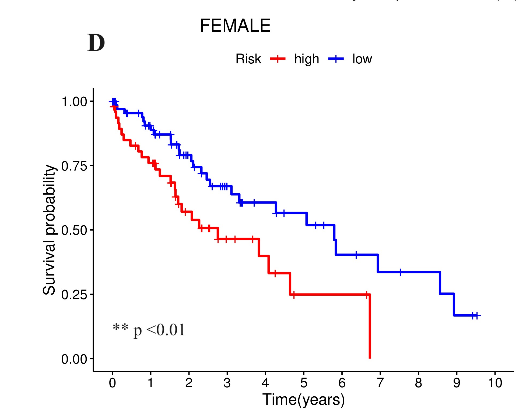


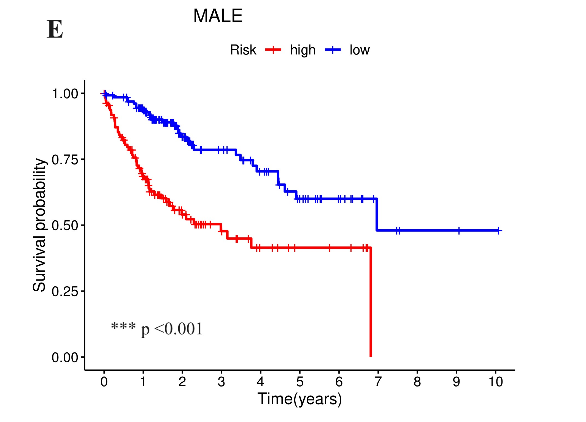

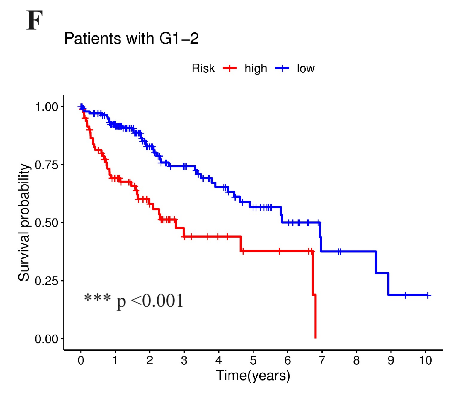

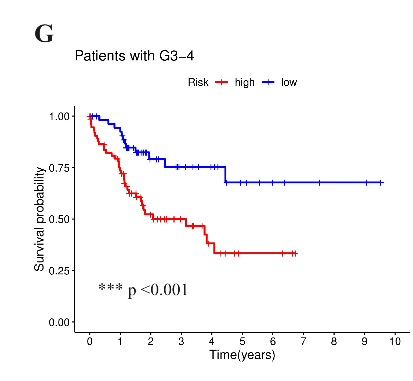


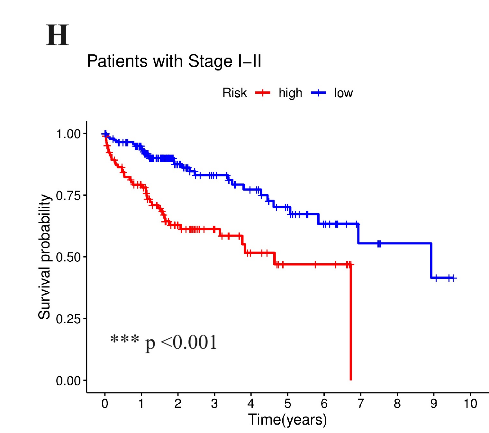

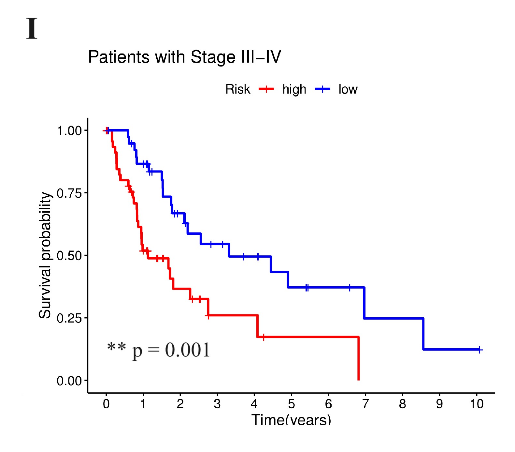


**Figure S3. Validation of the prognostic value of the disulfidptosis-related lncRNA (DRLs) signature model.** (A-B) Univariate Cox regression analysis of the clinical variables and DRLs risk scores model. (B) Area under the curve (AUC) about the 1-, 3- and 5-OS prediction of the DRLs prognostic signature. (C) C-index ROC curve of the risk score model and clinicopathological parameters. (D-I) Survival analyses of the risk score in different subgroups: Survival analyses of the risk score in different subgroups of various clinical factors: gender (female/male), pathological grade (1–2/3–4), and clinical stage (I–II/III–IV). (*P < 0.05, **P< 0.01, ***P < 0.001 ****P<0.0001, ns: no significance)
